# Retinal inner nuclear layer thinning is decreased and associates with the clinical outcome in ocrelizumab-treated primary progressive multiple sclerosis

**DOI:** 10.1007/s00415-022-11183-y

**Published:** 2022-06-01

**Authors:** Alessandro Miscioscia, Marco Puthenparampil, Silvia Miante, Marta Pengo, Francesca Rinaldi, Paola Perini, Paolo Gallo

**Affiliations:** 1 Department of Neuroscience DNS, School of Medicine, University of Padua, Via Giustiniani, 5, 35128 Padua, Veneto Region Italy; 2 Multiple Sclerosis Centre, University Hospital of Padua, Padua, Veneto Region Italy; 3 Present Address: Neurology Unit, Ospedale dell’Angelo, Mestre, Italy; 4 Present Address: Department of Molecular and Translational Medicine, University of Brescia, Brescia, Italy

**Keywords:** Optical coherence tomography, Primary progressive multiple sclerosis, Ocrelizumab, Neurodegeneration, Biomarker, Inner nuclear layer

## Abstract

**Background:**

Ocrelizumab was found to decrease brain atrophy rate in primary progressive multiple sclerosis (PPMS), but no data are currently available on the effect of ocrelizumab on retinal layer thicknesses in the PPMS population.

**Objective:**

To assess retinal layer changes in ocrelizumab-treated PPMS and test their possible application as biomarkers of therapy response.

**Methods:**

36 PPMS patients, treated with ocrelizumab for at least 6 months, and 39 sex- and age-matched healthy controls (HC) were included in a blind, longitudinal study. Spectrum-domain optical coherence tomography (SD-OCT) was performed at study entry (T0) and after 6 (T6) and 12 months (T12). At month 24 (T24), patients were divided into responders (no evidence of 1-year confirmed disability progression, 1y-CDP) and non-responders (evidence of 1y-CDP).

**Results:**

At T24, 23/36 (64%) patients were considered responders and 13/36 (36%) non-responders. At T0, peripapillary retinal nerve fiber layer (pRNFL) thickness, macular ganglion cell–inner plexiform layer (GCIPL) and inner retinal layer (IRL) volume were significantly lower in PPMS compared to HC (*p* = 0.001 for all comparisons). At T6 and T12, non-responders significantly differed in the inner nuclear layer (INL) thinning rate compared to responders (*p* = 0.005 at both time-points).

**Conclusions:**

Ocrelizumab significantly slows down INL thinning rate in PPMS responders. The longitudinal analysis of retina layer changes by means of OCT may be a promising prognostic test, and merits further investigations.

## Introduction

Multiple sclerosis (MS) is a chronic inflammatory, immune-mediated disease of the central nervous system, characterized by demyelination, axonal loss, and neurodegeneration. The initial presentation is mostly characterized by a relapsing–remitting (RRMS) disease course, often evolving in the secondary progressive (SPMS) phase, characterized by a gradual accumulation of disability independent of relapses [1]. Approximately 15% of patients have a primary progressive MS (PPMS) phenotype, defined by the gradual accrual of disability from disease onset, in absence of clinical relapses [2]. Neurodegeneration, observed as grey matter (GM) atrophy, is actually considered one of the major pathological substrates of disability in progressive MS (PMS).

Optical Coherence Tomography (OCT) is used in RRMS to analyze retinal nerve fiber layer (RNFL) and ganglion cell–inner plexiform layer (GCIPL) thicknesses as biomarkers of neurodegeneration [3, 4]. Indeed, GCIPL thinning was found (i) to be faster in RRMS with clinical or radiological evidence of disease activity [5], (ii) to mirror brain atrophy evolution [6], and (iii) to be influenced by disease-modifying drugs (DMD) [7]. A recent longitudinal OCT study showed faster thinning of inner nuclear layer (INL) and outer plexiform layer (ONL) in patients with progressive MS compared to age-matched RRMS and healthy controls (HC), indicating that these two retinal layers are worthy of investigation as possible biomarkers of neurodegeneration in the progressive forms of MS [8].

Important information on the clinical usefulness of biomarkers of neurodegeneration can be derived from their evaluation in patients under DMDs. Ocrelizumab, the only DMD approved for the treatment of PPMS, proved to slow down disability progression (as evaluated by the Expanded Disability Status Scale, EDSS) and brain volume loss in this form of MS compared to placebo [9].

No study has explored the effect of ocrelizumab on retinal layer atrophy in PPMS to date. Looking for biomarkers of neurodegeneration that could be easily used in clinical practice, we designed an explorative, longitudinal, blind OCT study aimed at analyzing retinal layer changes in a homogeneous group of ocrelizumab-treated PPMS patients.

## Methods

### Study design and participants

Between January 2019 and September 2021, 36 PPMS patients and 39 HC were consecutively recruited at the MS Center of the Veneto Region of the University Hospital of Padua. Inclusion criteria were (1) MS diagnosis according to the 2017 revised McDonald criteria [10], (2) primary progressive disease course [11], and (3) on-going therapy with ocrelizumab for at least 6 months. Exclusion criteria were (1) ophthalmologic pathologies (including iatrogenic optic neuropathy, diabetes, uncontrolled hypertension, glaucoma), (2) refractive errors (± 6 D), and (3) optic neuritis (ON) in the 6 months prior to enrollment or during follow-up. HC were recruited among the University Hospital staff and patients’ relatives and were matched for age and sex ratio with the PPMS cohort.

To avoid a possible pseudoatrophy effect due to the treatment, all patients were enrolled in the study at least 6 months after ocrelizumab initiation, after a re-baseline MRI scan was performed (T0). OCT was performed at T0 (enrollment in the study) and after 6 (T6) and 12 (T12) months. Clinical follow-up consisted in neurological examination and EDSS scoring every 6 months for 2 years (T24). OCT and clinical assessment were performed on the day of ocrelizumab infusion, before starting the pre-medication protocol.

At T24, patients were divided into non-responders = evidence of 1 year-confirmed disability progression (1y-CDP) and responders (non-1y-CDP). CDP was defined by an increase in EDSS score of 1.0 point in case of EDSS < 5.5 at baseline, or an increase of 0.5 if EDSS score was 5.5 or greater at baseline. OCT evaluation of HC was done only at study entry. OCT was performed by neurologists (AM, MPu) blinded to clinical parameters.

The study was approved by the Ethics Committee of University Hospital (Comitato Etico per la Sperimentazione, Azienda Ospedaliera Universitaria di Padova—Prot. N. 52,511) and carried out in accordance with the Declaration of Helsinki. Written informed consent was obtained by all the participants.

### Optical coherence tomography

Spectral Domain (SD)-OCT (Spectralis; Heidelberg Engineering version 1.7.0.0) was performed by a single certified neurologist in accordance with the APOSTEL recommendations [12]. Data on global peripapillary RNFL (pRNFL) thickness (μm) were obtained using a 12-degree ring scan (corresponding to a 3.5 mm diameter) manually placed around the optic disc. Data on the GCIPL, INL, outer plexiform-outer nuclear layer (OPNL), retinal pigment epithelium (RPE), inner retinal layer (IRL, including layers from RNFL to INL) volume (mm^3^) in the macular area were acquired using a macular volume scan centered on the fovea, and including a 6 mm ring area. Automated segmentation of OCT scans and quality control were performed. Scans violating international-consensus quality-control criteria (OSCAR-IB) [13] were excluded (*n* = 4 patients excluded due to poor OCT quality; *n* = 36 patients, of which 67 eyes, entered the final analysis). Data were collected and analyzed by investigators blinded relative to patients' clinical outcomes.

### MRI monitoring

At study entry (T0) and thereafter annually, all patients underwent brain and spinal cord magnetic resonance imaging (MRI) scans at our institution with a standardized 3-T MRI protocol (Ingenia, Philips Medical Systems, Best, The Netherlands). The core brain MRI protocol included 3D T1-weighted, 3D fluid-attenuated inversion recovery (FLAIR), 3D T2-weighted, 3D double inversion recovery (DIR), post-contrast gadolinium-enhanced T1-weighted imaging. The spinal cord MRI protocol included sagittal T1-weighted, short-time inversion recovery (STIR), axial T2-weighted through suspicious lesions, and post-contrast gadolinium-enhanced T1-weighted imaging. Details of this protocol have been published elsewhere [14].

### Statistical analysis

Statistical analyses were performed using SPSS 22.0 (StataCorp LP, College Station, TX, USA). Normality in measurements was tested graphically and using Kolmogorov–Smirnov test. Nonparametric tests were used for non-normal or skewed data and parametric tests for normally distributed data. Respectively, median (interquartile range) and mean (± standard deviation) are shown. Differences between groups were analyzed using the chi-squared test for categorical variables, the 2-tailed *t* test for parametric continuous variables, and the Mann–Whitney test for nonparametric continuous variables.

Differences for segmented retinal layer thickness or volume data between groups were analyzed using generalized estimating equations (GEE) as recommended [12]; these were adjusted for intrasubject intereye correlations and repeated measurements, and employed an exchangeable correlation structure.

A *p* value of 0.05 was accepted as statistically significant.

## Results

### Study population

At T24, 23/36 (64%) PPMS were classified as responders (non-1y-CDP), while 13 (36%) showed further clinical progression (1y-CDP), with a mean EDSS increase of 1.0 point (range 0.5–2.0). The two groups did not differ in gender (*p* = 0.335), age (*p* = 0.951), disease duration (*p* = 0.147) and EDSS at T0 (*p* = 0.766). Non-1y-CDP patients were observed to have a shorter length of treatment, but the difference did not reach significance (*p* = 0.076). No patient reported a previous history of ON or developed ON during the course of the study. 24/36 (67%) patients were treatment-naive before starting ocrelizumab, while 12 received immunosuppressive therapy several years before ocrelizumab initiation, with no clinical benefit. Namely, 5 (14%) had been treated with cyclophosphamide (median treatment duration 20.8 months, IQR 16–24) and 7 (19%) with azathioprine (median treatment duration 32.5 months, IQR 24–40). These patients were therapy-free for a mean period of 4.5 years before ocrelizumab and were equally distributed among non-1y-CDP and 1y-CDP (cyclophosphamide: 2 non-1y-CDP and 3 1y-CDP, chi-squared *p* = 0.231; azathioprine: 3 non-1y-CDP and 4 1y-CDP, chi-squared *p* = 0.197). Demographics and clinical characteristics of the study populations are shown in Table 1.

**Table 1:** Baseline Demographics and Clinical Characteristics

	HC	PPMS	HC vs PPMS *p*-value	PPMS responders	PPMS non-responders	R vs NR *p*-value
Subjects (eyes) (%)	39 (78)	36 (67)	–	23 (43) (64%)	13 (24) (36%)	–
Age, years, mean (SD)	53.3 (8.0)	51.5 (6.0)	0.139^a^	51.4 (6.5)	51.8 (5.0)	0.951^a^
Male, *n* (%)	22 (56.4%)	25 (69.4%)	0.281^b^	15 (65.2%)	10 (76.9%)	0.335^b^
EDSS, median (IQR)	–	6.0 (4.5–6.5)	–	5.5 (4.5–6.5)	6.0 (3.5–6.5)	0.766^c^
Disease duration, years, median (IQR)	–	11.0 (5.0–16.3)	–	10 (5.0–15.5)	15 (9.0–17.0)	0.147^c^
Length of Ocrelizumab therapy at baseline, months, median (IQR)	–	13.5 (9.5–21.8)	–	12.0 (7.0–19.5)	18.0 (12.0–25.0)	0.076^c^
Eyes with optic neuritis history, *n* (%)	–	0	–	–	–	–
Previous Cyclophosphamide therapy, *n* (%)	–	5 (14%)	–	2 (9%)	3 (23%)	0.231^b^
Previous Azathioprine therapy, *n* (%)	–	7 (19%)	–	3 (13%)	4 (31%)	0.197^b^

*HC* healthy controls, *PPMS* primary progressive multiple sclerosis, *R* responders (non-1y-CDP), *NR* non-responders (1y-CDP), *SD* standard deviation, *EDSS* expanded disability status scale, *IQR* inter-quartile range

Significance testing:

^a^2-tailed *t* test on means

^b^Chi-squared test

^c^Mann-Whitney test

### Baseline comparisons of retinal layer thicknesses

At T0, pRNFL thickness, GCIPL and IRL volume were significantly lower in PPMS compared to HC (*p* = 0.001 for all comparisons). A slightly lower GCIPL volume was observed in 1y-CDP compared to non-1y-CDP (*p* = 0.049). Albeit not significant, disease duration was slightly longer in 1y-CDP. All the other retina layers did not differ in thickness between HC and PPMS, as well as between non-1y-CDP and 1y-CDP patients. Data are summarized in Table 2.

**Table 2:** Baseline retinal layers thickness and comparisons between groups

	HC	PPMS overall	PPMS R	PPMS NR	HC vs PPMS	R vs NR
					*p*-value^a^	*p*-value^a^
Thickness, μm, mean (SD)						
pRNFL	102.57 (6.51)	93.00 (10.56)	94.37 (9.64)	90.17 (11.65)	0.001*	0.122
Volume, mm^3^, mean (SD)						
GCIPL	2.00 (0.14)	1.90 (0.18)	1.92 (0.18)	1.84 (0.18)	0.001*	0.049*
INL	0.98 (0.05)	0.96 (0.06)	0.96 (0.06)	0.96 (0.05)	0.058	0.826
OPNL	2.56 (0.15)	2.54 (0.17)	2.53 (0.15)	2.52 (0.16)	0.051	0.795
RPE	0.43 (0.02)	0.43 (0.06)	0.42 (0.03)	0.43 (0.08)	0.651	0.606
IRL	6.42 (0.28)	6.24 (0.38)	6.27 (0.35)	6.14 (0.36)	0.001*	0.161

*HC* healthy controls, *PPMS* primary progressive multiple sclerosis, *R* responders (non-1y-CDP), *NR* non-responders (1y-CDP), *SD* standard deviation, *pRNFL* peri-papillary retinal nerve fiber layer, *GCIPL* ganglion cell + inner plexiform layer, *INL* inner nuclear layer, *OPNL* outer plexiform + outer nuclear layer, *RPE* retinal pigment epithelium, *IRL* inner retinal layer (including layers from RNFL to INL)

^*^Indicates *p* < 0.05

^a^Generalized estimation equation model adjusted for inter-eye dependency

### Effects of ocrelizumab on longitudinal changes in retinal layer thicknesses in PPMS

**Fig. 1:**
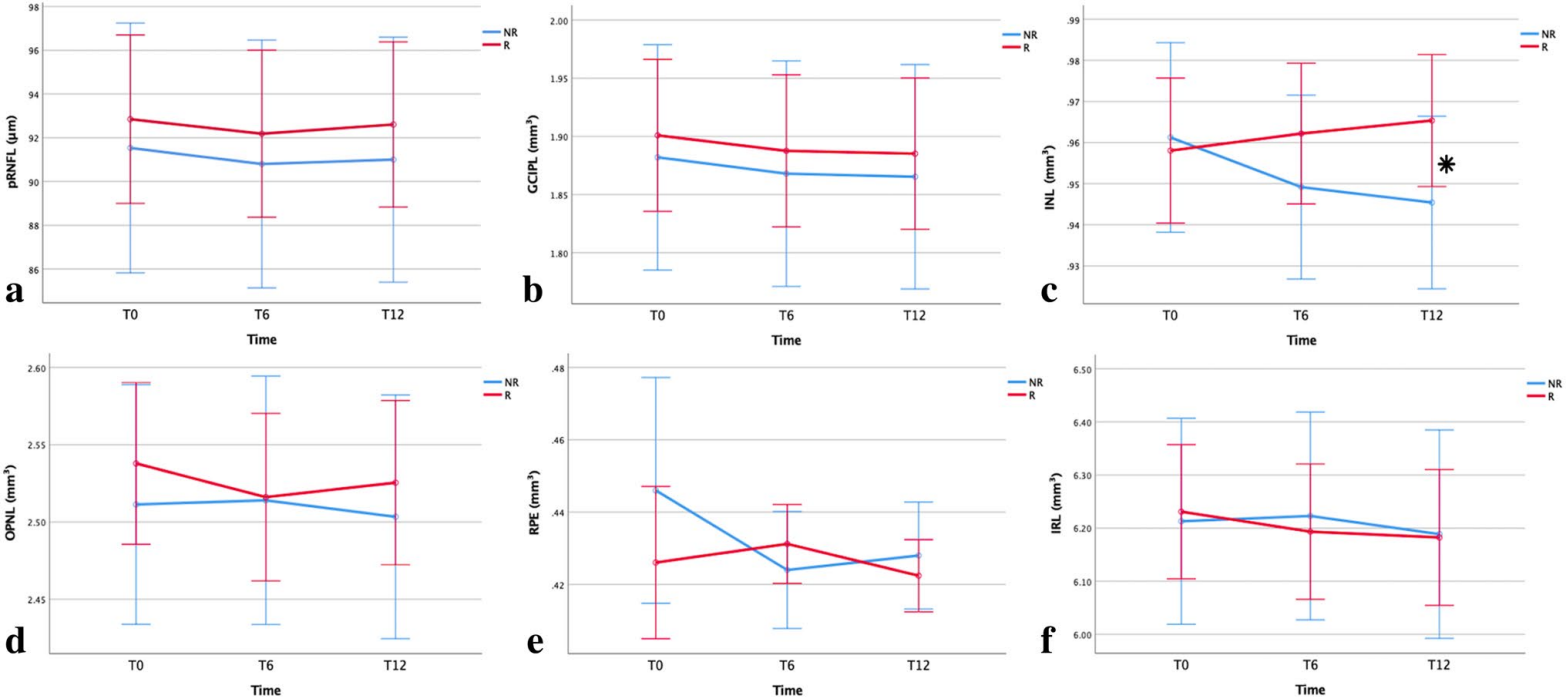
Longitudinal change in retinal pRNFL thickness and GCIPL, INL, OPNL, RPE, IRL volume with 95% confidence intervals (based on generalized estimating equation model) over time from baseline (T0) to 6 months (T6), and 12 months (T12), for PPMS responders (red line) and non-responders (blue line). * indicates *p* < 0.05. The INL volume (**c**) exhibited a stable-rising annual trend in ocrelizumab-responder patients (from 0.958 ± 0.009 to 0.965 ± 0.008 mm^3^), and it decreased in non-responders (from 0.961 ± 0.012 to 0.945 ± 0.011 mm^3^), reaching a significant difference (*p* = 0.005). No significant difference was observed between responders and non-responders for pRNFL (**a**), GCIPL (**b**), OPNL (**d**), RPE (**e**), and IRL (**f**)

Table 3 and Fig. 1 show the longitudinal changes observed in the retinal layers in non-1y-CDP versus 1y-CDP. From T0 to T6, INL volume remained substantially stable or even increased in non-1y-CDP (from 0.958 ± 0.009 to 0.962 ± 0.009 mm^3^; + 0.42%) and significantly (*p* = 0.005) decreased in 1y-CDP (from 0.961 ± 0.012 to 0.949 ± 0.011 mm^3^; − 0.26%). This finding was further confirmed at T12 (T0 vs T12: non-1y-CDP =  + 0.73%; 1y-CDP = − 0.69%; *p* = 0.005). We asked whether the increase in INL volume observed in some non-1y-CDP patients, a quite surprising finding, was responsible for reaching the significance. Thus, we performed a mock analysis assuming the stability of INL volume in non-1y-CDP throughout the study (T0–T6 INL volume change = 0 and T0–T12 INL volume change = 0) and found that the difference between 1y-CDP and non-1y-CDP was still significant at T6 (*p* = 0.013) and at T12 (*p* = 0.025). No difference in pRNFL thickness and GCIPL, ONPL, RPE, IRL volumes was observed between non-1y-CDP and 1y-CDP. Moreover, no new T2, T1-gad + or enlarging lesions were observed in both patient groups throughout the study.

**Table 3:** Comparison of change in retinal layer thickness between ocrelizumab responders vs non-responders

	PPMS	T0	T6	T12	T0–T6 percent change, mean (%)	T0–T12 percent change, mean (%)	R vs NR	
							T0–T6 *P*-value^a^	T0–T12 *P*-value^a^
Thickness, μm, mean (SE)								
pRNFL	R	92.85 (1.91)	92.18 (1.90)	92.61 (1.88)	– 0.73	– 0.26	0.301	0.167
	NR	91.53 (2.84)	90.80 (2.81)	91.00 (2.78)	– 1.11	– 0.58		
Volume, mm^3^, mean (SE)								
GCIPL	R	1.901 (0.032)	1.888 (0.032)	1.885 (0.032)	– 0.69	– 0.85	0.990	0.607
	NR	1.882 (0.048)	1.868 (0.048)	1.865 (0.048)	– 0.75	– 0.91		
INL	R	0.958 (0.009)	0.962 (0.009)	0.965 (0.008)	+ 0.42	+ 0.73	0.005*	0.005*
	NR	0.961 (0.012)	0.949 (0.011)	0.945 (0.011)	– 0.26	– 0.69		
OPNL	R	2.538 (0.026)	2.516 (0.027)	2.525 (0.026)	– 0.87	– 0.51	0.112	0.711
	NR	2.511 (0.039)	2.514 (0.040)	2.503 (0.039)	+ 0.12	– 0.32		
RPE	R	0.426 (0.010)	0.431 (0.005)	0.422 (0.005)	+ 1.17	– 0.95	0.267	0.652
	NR	0.446 (0.016)	0.424 (0.008)	0.428 (0.007)	– 5.19	– 4.21		
IRL	R	6.231 (0.063)	6.193 (0.063)	6.182 (0.063)	– 0.61	– 0.79	0.110	0.356
	NR	6.213 (0.096)	6.223 (0.097)	6.189 (0.097)	+ 0.16	– 0.39		

*PPMS* primary progressive multiple sclerosis, *T0* baseline, *T6* 6 month follow-up, *T12* 12 month follow-up, *R* responders (non-1y-CDP), *NR* non-responders (1y-CDP), *SE* standard error, *pRNFL* peri-papillary retinal nerve fiber layer, *GCIPL* ganglion cell + inner plexiform layer, *INL* inner nuclear layer, *OPNL* outer plexiform + outer nuclear layer, *RPE* retinal pigment epithelium, *IRL* inner retinal layer (including layers from RNFL to INL)

^*^Indicates *p* < 0.05

^a^Generalized estimation equations model adjusted for inter-eye dependency and repeated measurements

## Discussion

In this single-center, blind, longitudinal study, conducted in a quite homogeneous group of ocrelizumab-treated PPMS patients, we observed significant differences in INL volume changes between non-1y-CDP and 1y-CDP patients. Namely, EDSS increase in 1y-CDP was associated with INL atrophy progression, while EDSS stability paralleled with stabilization or increase of INL volume in non-1y-CDP. These results are in agreement with [8] post-mortem pathological findings [15] and in vivo observations suggesting that INL thinning can be a useful biomarker for tracking neurodegeneration in progressive MS [8]. Namely, Sotirchos et al. found a faster retinal atrophy, independent of age, in PMS compared to RRMS and HC, suggesting that INL and ONL measures may be novel biomarkers of neurodegeneration in PMS [8]. In this study, no DMD effect was observed on retinal parameters of PMS that included PPMS (with a mean disease duration of 7.5 years) and SPMS (with a mean disease duration of 15.5 years) treated with different DMDs (i.e. natalizumab, rituximab, daclizumab), and analysis for single treatment effect was not performed. Moreover, only 24 out of 60 (40%) PPMS were treated with DMD. Thus, our study significantly differs from that of Sotirchos et al. since it specifically assessed the effect of ocrelizumab in a quite homogeneous population of PPMS.

In agreement with literature data [3, 4, 8], we found that pRNFL thickness and GCIPL volume are significantly lower in untreated PPMS as compared to HC, further confirming that the analysis of retinal layers gives the chance of identifying atrophic changes that can be used as biomarkers of neuroaxonal damage. Moreover, the evidence that at baseline 1y-CDP patients, having a mean of 15 years of disease duration, had significantly lower GCIPL volume compared to non-1y-CDP patients, having 10 years of disease duration, strongly indicates that GCIPL atrophy associates with disease duration and parallels global brain and GM atrophy in progressive MS [6]. This observation further stresses the potential prognostic value of OCT examination.

Our findings point out the possible use of INL thinning as biomarker of DMD effect on the neurodegeneration in MS. Indeed, INL is composed of three neuronal cells (bipolar, horizontal, and amacrine cells) and two types of glial cells (Müller cells and microglia). Bipolar cells account for about 60% of all INL cells, and connect ganglion cells with photoreceptors [16]. Bipolar cells may be involved in trans-synaptic degeneration, especially in longstanding and progressive phases of the disease, when the ganglion cell layer (GCL) is known to be severely affected and atrophic. Thus, in parallel with the effect observed in the grey matter [9], the ocrelizumab-induced suppression of brain inflammation may also protect retinal neuronal cells from neurodegeneration that can result from both local subclinical inflammatory damage and trans-synaptic degeneration occurring along the visual pathway.

Our observations seem also in line with preclinical data obtained in animal models of brain inflammation mimicking MS. Indeed, the effect of anti-CD20 antibody treatment on GM pathology was investigated in human myelin oligodendrocyte glycoprotein-induced experimental autoimmune encephalomyelitis (huMOG-EAE) mice by means of serial brain volumetric MRI scans performed at baseline, 1 and 5 weeks post-huMOG-EAE induction and anti-CD20 treatment [17]. Compared to EAE controls, anti-CD20 therapy significantly reduced brain volume loss in the basal ganglia, isocortex and thalamus across all time-points longitudinally. Moreover, at cellular level, anti-CD20 therapy suppressed the percentage of proliferative nuclear antigen positive microglia. These findings suggested that anti-CD20 antibody treatment can delay grey matter degeneration by suppressing microglia activation and proliferation. Noteworthy, increased hyperreflective foci (HRF) considered proliferating microglia and suggestive of local subclinical inflammation have been demonstrated in the retina of RRMS patients [18]. Finally, a positive correlation between retinal microglia activation and GCIPL thinning was observed in EAE [19, 20], and a pivotal role of microglia in chronic neurodegeneration has been suggested by neuroimaging studies in MS patients [21, 22]. Studies that investigate the effect of ocrelizumab on microglia activation in MS using positron emission tomography (PET) with translocator protein (TSPO) radiotracers are currently in progress [23, 24].

With regard to the unexpected finding of an increasing trend in INL volume in responder patients, the underlying mechanism remains unclear, although an ocrelizumab-mediated effect on the local cellular network has to be taken into account. Since anti-CD20 may modulate microglia activities, and microglia-induced changes on Müller cells activation have been demonstrated [24], we may hypothesize an indirect effect on the physiological regulation of retinal blood flow and extracellular fluid homeostasis (the so-called glymphatic system) in responder patients [25]. Of course, further observations on a higher number of patients are needed to sustain this hypothesis.

The limited number of patients enrolled and the relatively short follow-up may constitute a major limitation of our study, which has to be considered explorative. However, we would like to stress that we studied a quite homogeneous cohort of PPMS patients, all diagnosed, treated and monitored in a single Centre with well-standardized techniques and methodologies. Moreover, a two-year clinical follow-up is in line with typical length of randomized controlled trials (RCTs). In addition, in our work we thoroughly took into account the current guidelines that recommend a re-baseline of the imaging parameters at least 6 months after therapy initiation to exclude residual disease activity due to treatment efficacy lag time [25]. Furthermore, a global brain volume loss has been described following the DMD initiation, the so-called ‘pseudoatrophy’ phenomenon [26–28], due to the resolution of brain inflammation, which results in edema resorption and decrease in number of inflammatory cells, included microglia [29]. Since no data are available to date on the possible pseudoatrophy phenomenon in the retina and to reduce possible bias due to the variability of brain inflammation at study entry, we enrolled patients who had completed at least three ocrelizumab infusions (i.e., 6 months after therapy initiation).

Taken all together, our findings suggest that ocrelizumab is effective in slowing down retinal atrophy in PPMS, and that INL volume change may represent a biomarker of ocrelizumab efficacy, pointing out the possible role of OCT in monitoring MS therapies.
